# Bumblebees Perform Well-Controlled Landings in Dim Light

**DOI:** 10.3389/fnbeh.2016.00174

**Published:** 2016-09-13

**Authors:** Therese Reber, Marie Dacke, Eric Warrant, Emily Baird

**Affiliations:** Department of Biology, Lund UniversityLund, Sweden

**Keywords:** bumblebee, *Bombus terrestris*, insect, landing, flight, vision, light intensity, behavioral adaptation

## Abstract

To make a smooth touchdown when landing, an insect must be able to reliably control its approach speed as well as its body and leg position—behaviors that are thought to be regulated primarily by visual information. Bumblebees forage and land under a broad range of light intensities and while their behavior during the final moments of landing has been described in detail in bright light, little is known about how this is affected by decreasing light intensity. Here, we investigate this by characterizing the performance of bumblebees, *B. terrestris*, landing on a flat platform at two different orientations (horizontal and vertical) and at four different light intensities (ranging from 600 lx down to 19 lx). As light intensity decreased, the bees modified their body position and the distance at which they extended their legs, suggesting that the control of landing in these insects is visually mediated. Nevertheless, the effect of light intensity was small and the landings were still well controlled, even in the dimmest light. We suggest that the changes in landing behavior that occurred in dim light might represent adaptations that allow the bees to perform smooth landings across the broad range of light intensities at which they are active.

## Introduction

To ensure a safe and smooth landing, a flying insect has to regulate its speed, modify its body posture and extend its legs in good time before making contact with the surface. Previous work has shown that, in a diverse range of insects, visual input is important for controlling these components of the landing behavior (flies: Wagner, 1982; Borst, 1986; Tammero and Dickinson, 2002; van Breugel and Dickinson, 2012; honeybees: Srinivasan et al., 2000; Baird et al., 2013; bumblebees and sweat bees: Baird et al., 2015). To make a smooth touchdown, these insects must therefore be able to reliably discriminate the surface from the background. As light levels fall, this task becomes more challenging because contrast discrimination becomes increasingly difficult. This is, in part, due to the low number of photons that are available and the fact that they arrive at the retina in a random and unpredictable way (Warrant and McIntyre, 1993). This random arrival of photons causes visual “noise” that degrades the reliability of vision (Rose, 1942; De Vries, 1943). Transducer noise (Lillywhite, 1977, 1981; Lillywhite and Laughlin, 1979; Laughlin and Lillywhite, 1982) and dark noise (Barlow, 1956) further add to the unreliability of vision at low light levels.

Despite the challenges imposed by the dim light on visually controlled behaviors, many insects fly and land successfully at night. Two of them are the nocturnal sweat bee *Megalopta genalis* and the Indian carpenter bee *Xylocopa tranquebarica* (Warrant et al., 2004; Kelber et al., 2006; Somanathan et al., 2008). This is remarkable, considering that bees have apposition compound eyes, an inherently inefficient design for collecting light. To adjust to their nocturnal lifestyle, these two bee species have evolved a number of optical adaptations (larger facets and wider rhabdoms), as well as neural adaptations (spatial summation) to make their visual systems more sensitive (Warrant, 1999; Greiner et al., 2004). Bumblebees also forage early in the morning and late in the evening (Spaethe and Weidenmüller, 2002), suggesting that they possess mechanisms to control their flight under low light conditions. Indeed, bumblebees temporally sum the signals in their photoreceptors (Reber et al., 2015). While such summation would provide a brighter image of the world, the increased visual processing time would limit the ability to detect fast visual motion. As an apparent attempt to reduce the effect of this trade-off, bumblebees fly slower as light levels fall (Reber et al., 2015). By decreasing their speed, the bees increase the likelihood of obtaining sufficient visual information to reliably see motion information and to use this to control their flight in dim light. This behavioral adaptation to dim light has also been observed in honeybees (Rose and Menzel, 1981), hornets (Spiewok and Schmolz, 2005) and moths (Sponberg et al., 2015).

While the behavior of bumblebees and honeybees during the final moments before touchdown has been described in great detail for bright light conditions (Evangelista et al., 2009; Reber et al., 2016), the effect of light intensity on the landing behavior of insects has received little attention. To the best of our knowledge, only one study (Baird et al., 2015) has addressed this by filming *B. terrestris*, while landing on a vertical surface at the nest under two different light intensities (190 and 19 lx). Interestingly, the bees extended their legs significantly later in the dimmer light condition, supporting the hypothesis posed by Reber et al. (2015) that bumblebees use temporal summation to increase light capture and improve visual reliability to control landing in dim light (since a longer integration time might delay the detection of the surface).

Since we now know that the landing behavior of bumblebees varies depending on the slope of the landing surface (e.g., they extend their legs further away from the surface when landing at horizontal compared to inclined surfaces; Reber et al., 2016), the aim of this current study is to obtain a deeper understanding of the effect of light intensity on the control of their landing behavior when approaching platforms of different orientations. To do this, we analyze the behavior of free-flying bumblebees (*B. terrestris*) landing on a flat (horizontally or vertically oriented) platform at four different light intensities (chosen to reflect natural light conditions ranging from sunrise or sunset on a clear day (600 lx) down to the middle of civil twilight (19 lx; Johnsen et al., 2006). We investigated the effect of light intensity on the approach trajectory and speed, as well as the position and duration of the hover phase (a short period preceding touchdown when the bees remain stationary in the air). There is reason to believe that the hover phase is affected by light intensity because previous work has suggested that this behavior has a strong visual component. It has been proposed that bees use the hover phase to visually inspect the pollen content (Zimmerman, 1982) or specular reflections from nectar droplets (Kevan, 1976) in flowers in order to evaluate if they are rewarding or not (Goulson et al., 2001). Landing performance is also characterized by the distance from the platform at which the bees extend their legs, the body posture at this moment in time, as well as the time between leg extension and first contact with the surface (time to contact, TTC).

## Materials and Methods

### Animals and Experimental Setup

**Figure 1:**
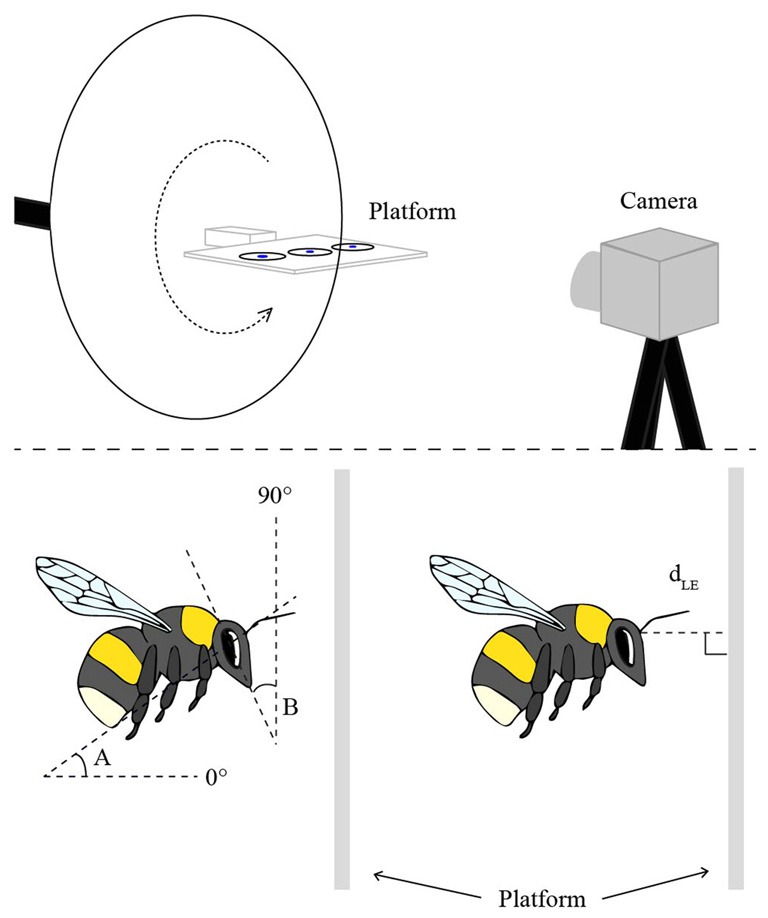
**The experimental setup (top), in which the bees were trained to visit a flat Perspex platform with three disks of white filter paper saturated with sugar solution.** Landings on the platform were filmed from the side with a high-speed camera. In order to film landings at different platform orientations, the platform was attached to a rotatable arm (dotted arrow). The schematic illustrations of the bees (bottom) indicate the two angular measurements of body posture (angles A and B) and the linear measurement of the distance to the platform at leg extension (d_LE_).

The experiments were performed with six hives of commercially bred bumblebees, *B. terrestris* (Koppert, Netherlands) in a flight cage (2.3 m long, 2.0 m high and 2.0 m wide, at 23°C) made from aluminum netting. The bees were trained to visit a feeder filled with sugar solution, placed on top of a flat, horizontally oriented, transparent Perspex platform (10 cm × 15 cm, 0.4 cm thick) that was attached to a tripod on a rotatable arm and positioned 1 m above the ground (Figure 1). Once the bees were regularly feeding from the “training feeder”, it was removed and they were instead presented with three disks of white filter paper (3 cm diameter), saturated with sugar solution and placed in a row along the center-line of the platform (Figure 1). A small disk of blue paper (1 cm diameter) was placed in the middle of each feeder disk to attract the bees to the center. Three disks were used to avoid an excess of bees at the same position. Bees that regularly visited the platform were individually marked with small plastic number plates on the back of their thorax. To discriminate the bees against the background in the camera images, a white cardboard disk (30 cm in diameter) was placed 10 cm behind the center of the platform.

### Experimental Procedure

Individual landings at the platform were filmed at 400 frames s^−1^ using a high-speed video camera (MotionBLITZ EoSens^®^ mini, Mikrotron GmbH, Germany; image resolution: 1280 × 600 pixels; F-number = 1.4 (focal length = 8 mm, aperture diameter = 5.7 mm) placed 16 cm away from the center of the platform. The platform was rotated to two different orientations measured from the horizontal plane: 0° and 90°, at four different light intensities: 19 lx (1.62 × 10^13^ photons/cm^2^/s), 60 lx (4.30 × 10^13^ photons/cm^2^/s), 190 lx (1.76 × 10^14^ photons/cm^2^/s), and 600 lx (5.64 × 10^14^ photons/cm^2^/s; as measured at the platform [lx measurements: Hagner ScreenMaster, B. Hagner, Sweden; photons/cm^2^/s measurements: QE65000, Ocean Optics Inc., Dunedin, FL, USA]). Light was provided by two dimmable fluorescent lamps (flicker frequency >20 kHz, BIOLUX^®^, OSRAM GmbH, Germany) attached to the ceiling of the cage. These lamps have a broad daylight spectrum with peaks at 450 nm, 545 nm, and 610 nm, as well as in the UV (UVC <2 μW/klm, UVB <20 mW/klm, UVA <150 mW/klm). To achieve the darkest light level (19 lx), the lamps were further dimmed with neutral density filters (210 0.6 ND, LEE Filters, UK). In the darker conditions, infrared illumination (peak wavelength: 850 nm, which is invisible to the bees) was used to discern the bees in the videos. Before each 1-h long experimental trial, the bees were given 30 min to adapt to the new light intensity. All experiments were conducted between 08:00 and 14:00, when the bees were most active. To avoid any circadian influence on the landing behavior, the trials were pseudo-randomized throughout the experiment. Only bees that landed alone on a feeder disk were used in the analysis to avoid any disturbance from other bees.

### Analysis

#### The Hover Phase, Time to Contact, and Flight Speed

To visualize the flight trajectories and analyze the bees’ position over time, seven individual landings at each platform tilt (and light intensity) were manually digitized with a custom made tracking software in Matlab (Mathworks Inc., Natick, MA, USA). These trajectories allowed us to examine the distance between the bee and the platform during the hover phase (the short period just before touchdown, during which the bees were almost [±1 mm] stationary in the air), the duration of the hover phase, the TTC (defined as the time between start of leg extension and touchdown), and the mean flight speed (just before the hover phase, as well as just before touchdown). For analysis of the distance between the bee and the platform during the hover phase, a mean value of this distance at the start and at the end of the hover phase was used. Flight speed was calculated by dividing the two-dimensional distance each bee traveled between two successive frames by the time between two frames (1/80 s). The frame rate used in the speed calculation was 80 frames s^−1^ because the bees were tracked every five frames. Flight speed was further analyzed at two different time points: 10 ms before the hover phase, and 10 ms before touchdown. At each time point, a mean value of the flight speed over 25 ms was used.

#### Body and Head Orientation and the Distance to the Platform at Leg Extension

The body and head orientations and the distance of the bee to the platform were determined in the frame (2.5 ms) prior to the frame in which the bee started to extend its legs in preparation for contact with the platform. Only bees that landed in side view (or nearly so) were used in the analysis. In the selected frame, for each bee, two angles were measured (using Screen Protractor 4.0, Iconico, Inc., New York, NY, USA): angle A between the long axis of the body and the horizontal plane, and angle B between the vertical plane and a line drawn from the top of the head through the tip of the mouth (for schematic illustrations of these measurements, see Figure 1). The perpendicular distance between the base of the antennae and the landing surface (d_LE_) was also measured for each frame (using ImageJ 1.47v, Wayne Rasband, National Institutes of Health, Bethesda, MD, USA; Figure 1).

### Statistics

All statistical tests were performed using SPSS (IBM SPSS Statistics 20, USA). Two-way ANOVAs were conducted for each data set to examine if there was an interaction between platform tilt and light intensity on all the different measurements in this study. If a significant interaction was found, a one-way ANOVA was conducted for each platform tilt to examine the effect of light intensity. If no interaction was found, *post hoc* tests (LSD) were performed on the effect of light intensity.

## Results

### Distance to the Platform During the Hover Phase and the Duration of the Hover Phase

**Figure 2:**
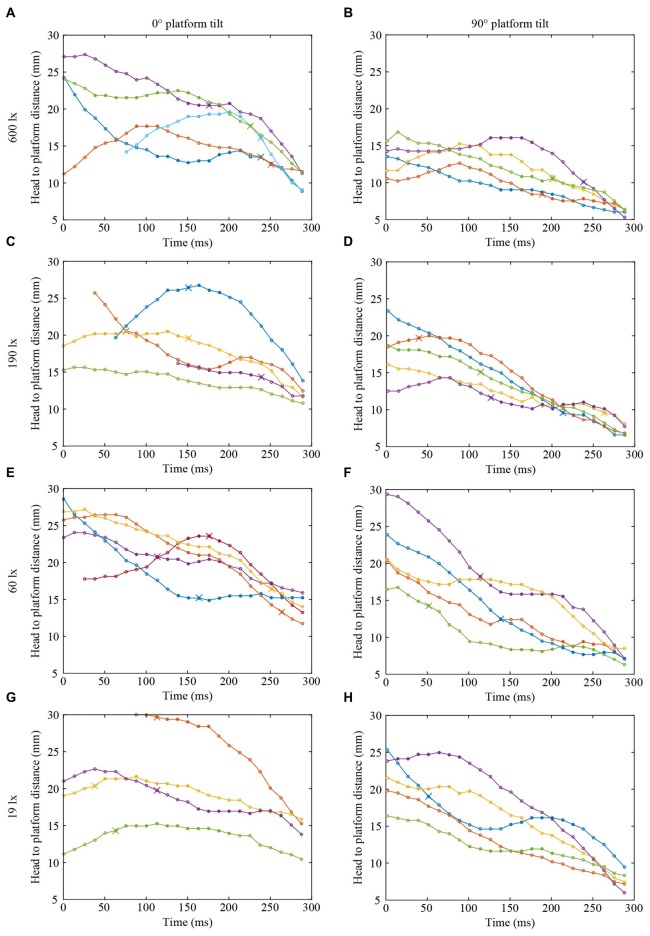
**Flight trajectories of landing bumblebees at different light intensities (A,B) 600 lx, (C,D) 190 lx, (E,F) 60 lx, (G,H) 19 lx, and platform tilts (A,C,E,G) 0°, (B,D,F,H) 90°.** For clarity, only four or five trajectories are shown in each condition. *Circles* represent the distance between the base of the antennae and the platform surface. *Filled circles* represent the time spent in the hover phase and *crosses* indicate the timing of leg extension. Trajectories without a cross represent landings where leg extension occurred outside of the graph window, except the blue line in **(B)** where leg extension occurred after contact. The last point in each graph indicates when the bee contacted the surface. In each graph, different colors represent different bees, but the same color in different graphs does not represent the same bee.

**Figure 3:**
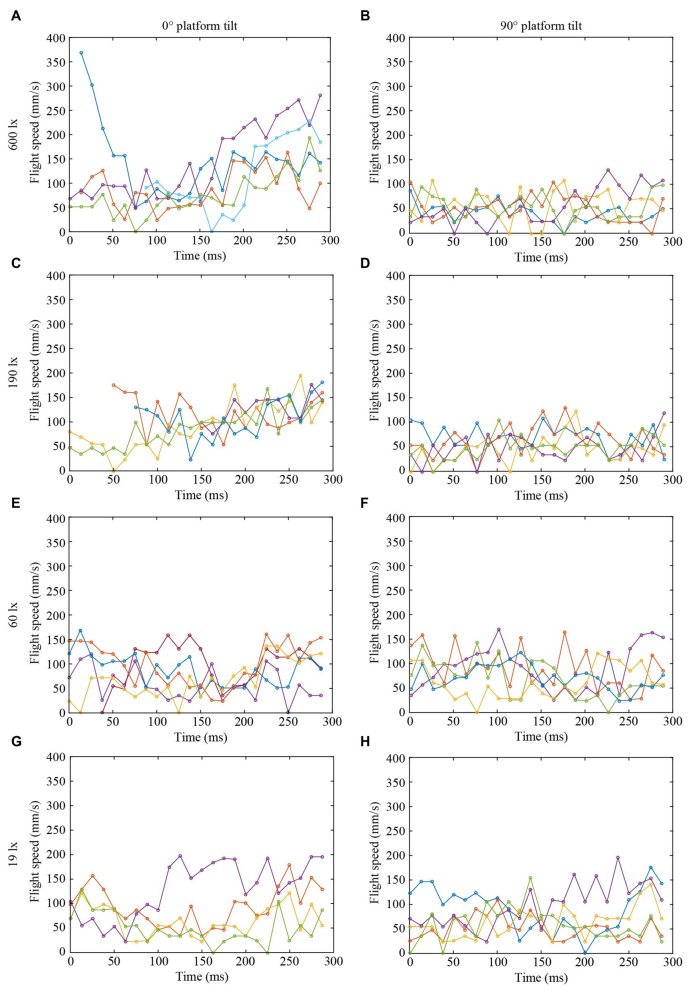
**Flight speed of landing bumblebees (same color in each graph in Figures 2, 3 represent the same bee) at different light intensities (A,B) 600 lx, (C,D) 190 lx, (E,F) 60 lx, (G,H) 19 lx, and platform tilts (A,C,E,G) 0°, (B,D,F,H) 90°**.

**Figure 4:**
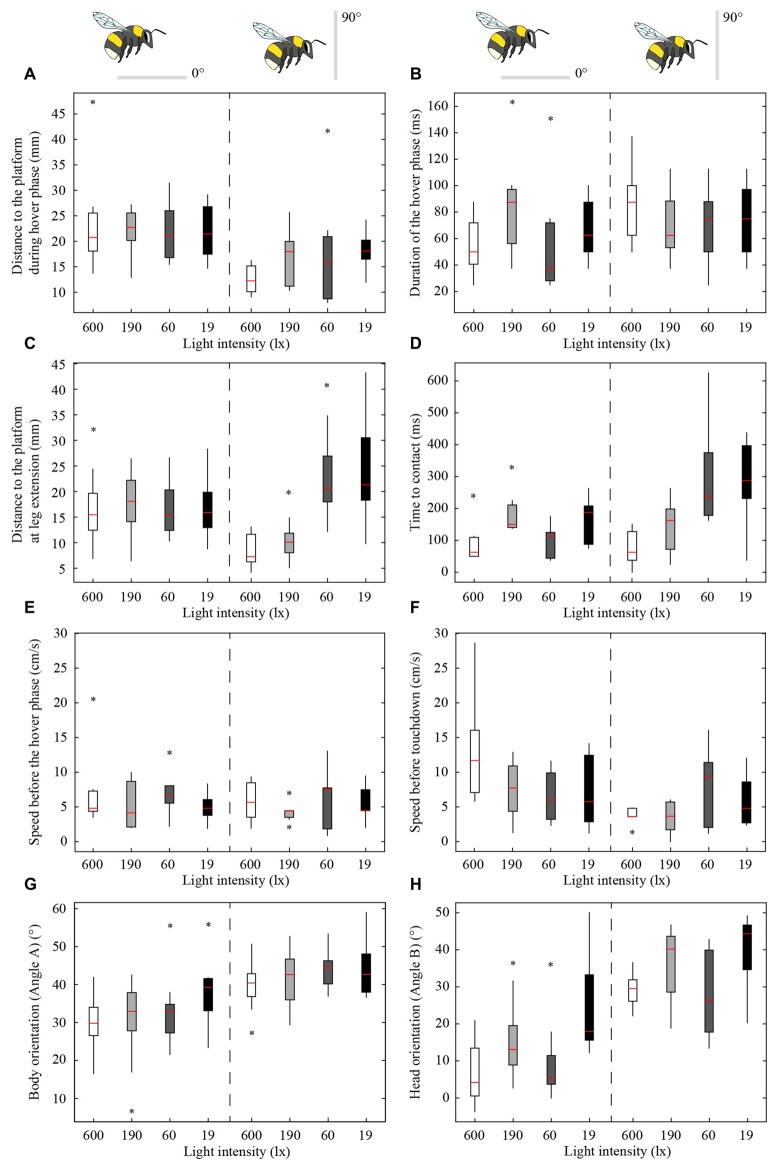
**Effect of light intensity and platform tilt on the distance to the platform during the hover phase (A) duration of the hover phase (B), distance to the platform at leg extension (C) time to contact (TTC; D) speed before the hover phase (E) speed before touchdown (F) body orientation (G) and head orientation (H) of bumblebees landing on a flat platform at two different orientations (0° and 90°) and at four different light intensities (19, 60, 190 and 600 lx).** The schematic illustrations of the bees and the platforms indicate the orientation of the platform. The edges of the boxes denote the 25th and 75th percentiles, the central red mark is the median, the whiskers extend to the most extreme data points, and the black stars indicate outliers. For the number of analyzed individuals at each platform tilt and light intensity, see Table 2.

Figures 2, 3 show the position and speed, respectively, of landing bumblebees during the last 300 ms before touchdown. During this time interval, the bees hovered for a short period before extending their legs (marked with X in Figure 2). To investigate the ability of the landing bees to assess the distance to the platform in dim light, we examined the distance between the bee and the platform during the hover phase at two different platform orientations (0° and 90°) and at four different light levels (19, 60, 190 and 600 lx). We found that the distance was significantly shorter for the vertical platform than for the horizontal platform, i.e., the bees hovered closer to the vertical platform (Figure 4A, Table 1). However, we found no significant effect of light intensity on hover distance (Table 1), indicating that, even in dim light, bumblebees are able to perform well-controlled landings. One way that they might achieve this is to increase the time that they spend in the hover phase, a strategy that would allow their visual system time to collect more light and increase visual reliability before contacting the surface. We therefore analyzed the duration of the hover phase preceding touchdown, but found that this was not significantly affected by either platform tilt or light intensity (Figure 4B, Table 1). Thus, hover phase duration does not appear to be modified to provide a gain in visual reliability in dim light.

**Table 1:** **Statistical details (*test, factor, F-value* and *P-value*) for measurements of landing performance of *B. terrestris* at platforms of different tilt (0° and 90° under different light intensities (ranging from 600 lx down to 19 lx)**.

Measurement	Statistical test	Factor	*F*	*P*	LI (lx)	Tilt (°)
Distance to platform	Two-way ANOVA	Tilt * LI	0.90	0.45		
during hover phase	”	Tilt	10.70	<0.01		
	”	LI	0.20	0.90		
Duration of	Two-way ANOVA	Tilt * LI	1.19	0.32		
hover phase	”	Tilt	0.92	0.34		
	”	LI	0.49	0.69		
	Two-way ANOVA	Tilt * LI	12.24	<0.001		
	One-way ANOVA	LI	0.12	0.95		0
Distance to platform	”	”	25.11	<0.001		90
at leg extension	*Post hoc* (LSD)	LI		0.42	600–190	90
	”	”		<0.001	600–60	”
	”	”		0.52	60–19	”
	Two-way ANOVA	Tilt * LI	4.71	<0.01		
	One-way ANOVA	LI	3.35	0.036		0
	”	”	4.94	0.01		90
Time to contact	*Post hoc* (LSD)	LI		0.020	600–190	0
from leg extension	”	”		0.96	600–60	”
	”	”		0.07	60–19	”
	”	”		0.37	600–190	90
	”	”		<0.01	600–60	”
	”	”		0.85	60–19	”
Flight speed prior	Two-way ANOVA	Tilt * LI	0.24	0.87		
to hover phase	”	LI	0.88	0.46		
	Two-way ANOVA	Tilt * LI	3.18	0.032		
Flight speed prior	One-way ANOVA	LI	2.09	0.13		0
to touchdown	”	”	1.98	0.14		90
	Two-way ANOVA	Tilt * LI	0.65	0.59		
Body angle (Angle A)	”	Tilt	46.40	<0.001		
	”	LI	3.25	0.025		
	*Post hoc* (LSD)	LI		<0.001	600–19	
	Two-way ANOVA	Tilt * LI	0.86	0.47		
	”	Tilt	121.03	<0.001		
Head angle (Angle B)	”	LI	14.62	<0.001		
	*Post hoc* (LSD)	LI		<0.01	600–190	
	”	”		<0.001	190–19	
	”	”		0.84	600–60	

*”=Same as above. For details on measurements, see “Materials and Methods” Section*.

**Table 2:** **Number of different individuals analyzed for measurements of landing performance of *B. terrestris* under different light intensities (ranging from 600 lx down to 19 lx) and two tilts of the landing platform (0° and 90°)**.

LI (lx)	Tilt (°)	d_HP_	dur_HP_	d_LE_	TTC	νPHP	νPTD	A,B
600	0	7	7	16	7	7	7	16
	90	7	7	16	6	7	7	16
190	0	7	7	16	7	6	7	16
	90	7	7	16	5	7	7	16
60	0	7	7	15	7	6	7	15
	90	7	7	16	7	7	7	16
19	0	7	7	7	7	7	7	7
	90	7	7	16	7	7	7	16

*d_HP_, distance to the platform during the hover phase; dur_HP_, duration of the hover phase; d_LE_, distance to the platform at leg extension; TTC, time to contact; *V*_PHP_, mean flight speed prior to the hover phase; *V*_PTD_, mean flight speed prior to touchdown; A, body orientation; B, head orientation. For details on measurements, see “Materials and Methods” Section*.

### Distance to the Platform at Leg Extension, Time to Contact and Flight Speed

Another way to facilitate landing in dim light might be to extend the legs earlier—a safety measure that would allow the bees to account for errors in estimating the distance to the platform. To investigate if bumblebees employ this strategy, we measured the distance from the platform at which leg extension was initiated (for examples of flight trajectories with the timing of leg extension indicated, see Figure 2). We found that the bees extended their legs significantly further away from the platform as light intensity decreased from 600 lx to 60 lx in preparation for a touchdown on the 90° platform, while light intensity had no measurable effect when the platform tilt was 0° (Figure 4C, Table 1).

To further investigate the effect of light intensity on the timing of leg extension, we also analyzed the TTC, defined as the time between leg extension and first contact with the surface. We found that light intensity affected TTC, but that the effect was different for the two platform tilts (Figure 4D, Table 1). At 0°, we found a significant increase in TTC as the light intensity decreased from 600 lx to 190 lx. However, we found no difference between 600 lx and 60 lx, or between 600 lx and 19 lx (Table 1). At 90°, TTC increased in a more linear fashion as light levels fell (Table 1). This could either be an effect of the earlier leg extension observed above (an extension of the legs further away from the platform will result in a longer TTC if the bees are moving at the same speed) and/or due to a decrease in the approach speed of the bees. To investigate if the bees reduce flight speed during landing in dim light, we measured the mean flight speed of the bees just before the hover phase (*v*_BHP_), as well as just before touchdown (*v*_BTD_; to see how flight speed varied over time during landing, see Figure 3), but found no effect in either case (Figures 4E,F, Table 1).

### Body and Head Orientation at Leg Extension

To investigate the effect of light intensity on landing posture, we analyzed the body and head orientation of the bees just before leg extension. The angle between the body and the horizontal plane (angle A) was significantly larger for the vertical platform than for the horizontal platform, i.e., the bees tilted their body more vertically when landing on the vertical surface (Figure 4G, statistical details in Table 1). Similarly, as light levels decreased from 600 lx to 19 lx, the body angle significantly increased when landing at both platform tilts (Figure 4G, Table 1), i.e., the bees flew in a more upright posture as light levels fell.

The angle between the head and the vertical plane (angle B) was also larger for the vertical platform than for the horizontal platform, indicating that the bees approached the vertical platform with their head in a more horizontal orientation (Figure 4H, Table 1). Furthermore, as light intensity decreased, the head angle also increased significantly except when comparing the orientations at 600 lx and 60 lx (Figure 4H, Table 1). As with body orientation, the effect of light intensity on head orientation was the same for both platform tilts (Table 1).

## Discussion

### Light Intensity does not Affect the Hover Phase

Given that the purpose of the hover phase is to mediate visual tasks (Kevan, 1976; Zimmerman, 1982), it is also reasonable to assume that the initiation of the hover phase itself is controlled using visual cues. If bumblebees do indeed rely on vision to control the distance from the platform at which they initiate the hover phase, then we might expect that this will be affected in dim light due to the reduced reliability of visual information. Surprisingly, the bees hovered at the same distance from the platform regardless of light level (Figure 4A, Table 1), although they hovered closer to the vertical platform (16 ± 7 mm, mean ± SD) than to the horizontal platform (22 ± 7 mm). This result is interesting for two reasons. First, it suggests that the bees regulate their hover distance based on the orientation of the platform, most likely due to the different approach angles of the bees required in each case. Second, the lack of effect of light intensity suggests that the visual system of the bumblebees is sensitive enough to accurately assess the distance to the platform at light levels as low as 19 lx. One strategy that would enable the bees to increase the reliability of their distance estimation in dim light would be to increase the duration of the hover phase. However, we found that hover duration remains constant, irrespective of changes in light intensity or platform tilt. It is also possible that hover distance is regulated by non-visual sensory information, such as mechanosensory cues coming from the air flow from the wings being deflected off the platform (it is also important to note that no part of the bee was ever observed to be in contact with the platform during the hover phase). It is not possible from our data to determine exactly what cues bumblebees use to control hover distance, but it is nonetheless remarkable that they are capable of making such fine distance measurements even in dim light.

Interestingly, during landings on the vertical platform and at the two lower light levels (60 and 19 lx), the bees were found to extend their legs before they initiated their hover phase (compare Figures 4A,C). This indicates that the hover phase and leg extension are not dependent upon each other, although our data suggest that they are both clearly important components of the landing behavior of bumblebees.

### Light Intensity Affects Leg Extension and Time to Contact But Not Approach Speed

Light intensity affected the distance at which bees extended their legs before landing when the platform was vertical, but not when it was horizontal. As light levels decreased, the bees initiated leg extension further away from the vertical platform (from 8 mm to 24 mm from 600 lx to 19 lx) but at a constant distance of 16–18 mm from the horizontal platform (Figure 4C, Table 1). The reason for this difference is unclear. It may be that, because the bees approach the vertical platform “head on”, they are more careful when approaching it in the dark. This earlier leg extension might be a safety mechanism to compensate for possible errors in the estimate of the distance to the vertical platform. In contrast, such a mechanism would not be necessary when landing on the horizontal platform because the bees are not in danger of flying directly into it. We have recently shown that, as light intensity decreases, bumblebee photoreceptors sum photons over an increasingly longer time to enhance the sensitivity of their eyes (Reber et al., 2015). A consequence of this strategy is that fast-moving objects become blurred or disappear completely from the visual field of the bee (Warrant, 1999) and stationary objects that are approached (such as the platform) are detected with a longer time delay. By extending the legs further away from the vertically oriented platform, bumblebees might minimize the risk of crashing, an event that would occur if they do not initiate landing in time. Such a response would not be necessary when the platform is horizontal, as the bees are not approaching the surface head on.

As with the leg extension distance, light intensity had an effect on TTC for bees approaching the vertical platform (the mean TTC for the vertical platform significantly increased from 73 ms to 286 ms from 600 lx to 19 lx), but not for the horizontal platform (TTC remained constant around 134 ms across all light levels). Because TTC is affected by both leg extension distance and speed, the increased TTC for the vertical platform may not only be due to an increased distance, but also due to a lower approach speed. However, the approach speed of the bees just before the hover phase (6 ± 3 cm s^−1^, mean ± SD) and just before touchdown (7 ± 5 cm s^−1^) did not differ across the light levels tested. Thus, the longer TTC recorded in dim light appears to simply be an effect of the greater distance at which leg extension is initiated.

The fact that the bees approached the platform at the same speed, irrespective of light level, provides some indication that their visual system is sensitive enough to perform well-controlled landings without adapting their landing flight speed, as they do when flying along experimental tunnels (Reber et al., 2015). However, an important difference between landing and cruising is that, when landing, the bees fly at a much lower speed (6 cm s^−1^ when landing vs. 89 cm s^−1^ when flying along a 30 cm wide tunnel). It is possible that, in the final touchdown phase of landing, the bumblebees have already reduced their speed to the lower limit of their range so that reducing it further provides no direct advantage. It is also possible that other adaptations to dim light such as the changes in distance of leg extension and body posture are sufficient for ensuring a safe touchdown without requiring a change of speed.

It is interesting to note that, in a recent study, Baird et al. (2015) also showed that light intensity had an effect on the TTC of bumblebees. In this earlier case, TTC decreased with light intensity, whereas here we observed the opposite effect. It is not clear why light intensity had a different effect in the two studies, but there are several important differences that might have played an important role. First, the behavioral context differed. In the present study, the bees were landing on a food source, while in Baird et al. (2015) they landed at the entrance of their nest. Hoverflies have been observed to approach flowers in a careful and meandering way if the goal is to feed but, if a possible mate is sitting on the surface of the flower, the fly instead accelerates towards the flower in a more direct way in the hope of courtship (Collett and Land, 1975). In a similar way, bees approaching a food source might do so more slowly and carefully than when approaching their nest. While an attempt to feed most likely includes a visual evaluation of the “flower” (Kevan, 1976; Zimmerman, 1982), landings at a familiar nest can be more direct and determined. Another factor that may have influenced the effect of light intensity on landing behavior in the two studies is the visual appearance of the platform. Baird et al. (2015) presented the bees with a 10 cm diameter disk that displayed a black and white concentric ring pattern, while in the present study, they were presented with a 1 cm diameter blue dot in the center of a 3 cm diameter white disk. These differences raise important questions about the effect of behavioral context and visual information on the landing behavior of bumblebees that will be addressed in future investigations.

### Vision Plays a Role in The Control of Body and Head Orientation During Landing

Changes in light intensity affected both the body and head orientation of the landing bumblebees. As light intensity decreased, the bees oriented their bodies more vertically and their heads more horizontally with respect to the platform (Figures 4G,H, Table 1). Blowflies walking in complete darkness also tilt their heads and bodies more vertically in comparison to walks in bright light (Kress and Egelhaaf, 2012). This allows them to use their front legs as tactile probes by stretching them out in front of the body. Although the effect of light intensity on the head and body orientation of bumblebees was relatively small (20° or less in most cases), it may represent a similar safety mechanism that allows the bees to make a precise leg-first touchdown, even when landing in very dim light. In fact, under the conditions presented in this study, not a single bee was observed to crash into the platform. The observation that body and head orientation are affected by changes in light intensity strongly suggests that these aspects of body posture are controlled by visual information, although exactly what visual information and how it is being used remains unclear.

## Conclusion

Here, we have shown that light intensity has an effect on the timing of leg extension and the body posture of bumblebees landing on a flat platform at two different orientations (0° and 90° relative to the horizontal plane) and at four different light intensities (ranging from 600 lx down to 19 lx). However, the changes in body posture are relatively small compared to the change in platform tilt (90°), and the landings are still well controlled even in dim light. The earlier leg extension combined with the changes in body posture might be a behavioral adaptation to dim light that allows the bees to make well-controlled landings across the broad range of light intensities at which they fly and forage. The consistent observation that light intensity affects the behavior of bumblebees in the final moments before touchdown provides a strong indication that landing is under predominantly visual control, although exactly what visual information is used and how remains unclear and will be the topic of future investigations.

## Author Contributions

TR performed the experiment and data analysis, and wrote the first draft of the manuscript. All authors (TR, MD, EW, EB) participated in the design of the experiment, the interpretation of the data and the final version of the manuscript.

## Funding

This work was supported by the Swedish Research Council Formas [229-2010-829], the Swedish Foundation for Strategic Research [FFL09-0056], and the Swedish Research Council [2011-4701, 2014-44295-114711-25].

## Conflict of Interest Statement

The authors declare that the research was conducted in the absence of any commercial or financial relationships that could be construed as a potential conflict of interest.
